# Associations of Serum Magnesium With Insulin Resistance and Testosterone in Women With Polycystic Ovary Syndrome

**DOI:** 10.3389/fendo.2021.683040

**Published:** 2021-06-23

**Authors:** Xi Luo, Wang-Yu Cai, Hong-Li Ma, Jing Cong, Hui Chang, Jing-Shu Gao, Wen-Juan Shen, Yu Wang, Xin-Ming Yang, Xiao-Ke Wu

**Affiliations:** ^1^ Department of Obstetrics and Gynecology, The Second Affiliated Hospital of Zhejiang Chinese Medical University, Hangzhou, China; ^2^ The Fourth Affiliated Hospital, Zhejiang University School of Medicine, Yiwu, China; ^3^ Department of Obstetrics and Gynecology, First Affiliated Hospital, Heilongjiang University of Chinese Medicine, Harbin, China; ^4^ Heilongjiang Province Hospital, Harbin, China

**Keywords:** Serum magnesium, PCOS, insulin resistance, testosterone, nutrition

## Abstract

**Objective:**

This article aimed to investigate whether serum magnesium is associated with insulin resistance index and testosterone level in women with polycystic ovary syndrome (PCOS).

**Materials and Methods:**

Overall 1000 women with PCOS were enrolled in a randomized controlled trial and a cross-sectional analysis of the association of serum magnesium with glucose metabolism markers and testosterone was performed. Serum magnesium, glucose metabolism markers and testosterone were measured. Insulin resistance was evaluated by homeostatic model assessment of insulin resistance (HOMA-IR) and quantitative insulin-sensitivity check index (QUICKI). Multivariable linear regression and logistic regression models were used to estimate the association between serum magnesium, insulin resistance and testosterone.

**Results:**

In comparative analyses, women with higher quartile of serum magnesium had significantly lower fasting glucose, HOMA-IR and testosterone. Multiple linear regression showed serum magnesium was independently negatively associated with insulin, glucose, HOMA-IR, testosterone and positively associated with QUICKI (P for trend <0.05) after adjusting confounding covariates. Logistic regression showed serum magnesium in quartile 1 and 2 were independently associated with insulin resistance status (Quartile 1: OR: 2.15, 95%CI: 1.35-3.40, P = 0.001; Quartile 2: OR: 1.90, 95%CI: 1.20-3.02, P = 0.006), while quartile 1 was marginally associated with hyperandrogenemia status (Quartile 1: OR: 1.45, 95%CI: 0.99-2.11, P = 0.055) after adjusting confounding covariates.

**Conclusion:**

The current findings suggest that lower serum magnesium was associated with aggravated insulin resistance and higher testosterone levels among women with PCOS.

## Introduction

Polycystic ovary syndrome (PCOS) is one of the most common reproductive and endocrinological disorders among women in child-bearing age (1). It presents various features including oligomenorrhea, hyperandrogenism, polycystic ovaries, insulin resistance (IR), and infertility and so on (2, 3). The clear cause of PCOS is unknown, but IR and hyperandrogenemia are thought to be important etiologies (4). It is generally acknowledged that nutrients disorder may also compromise the integrity of the endocrinological and reproductive performance (5–7). Magnesium plays an important role in glucose uptake, carbohydrate metabolism and energy transport (8). For example, lower magnesium in diet and blood were both believed to play a role in glycemic control and possibly affect the development of type 2 diabetes (9, 10). Magnesium intake was also negatively correlated with testosterone in women with PCOS (11).

However, studies exploring the role of serum magnesium in glucose metabolism and testosterone in women with PCOS are limited. Serum magnesium concentration appears to be declined in women with PCOS in some studies (12, 13). One systematic review found that serum Mg concentrations appear to be declined in overweight or obese women with PCOS (13). PCOS women with IR exhibited significantly lower serum levels of magnesium than PCOS women without IR (14). One randomized controlled trial of 60 women with PCOS found that magnesium supplementation did not influence serum lipid profiles and glycemic indicators but lead to marginally more decrease of testosterone levels compared to placebo (15). Larger trials investigating the association between serum magnesium, IR and testosterone in women with PCOS are still needed.

Knowledge in the association between serum magnesium, IR and testosterone in women with PCOS could provide more insight into the pathogenesis and management of PCOS. The present study aimed to examine the association of serum magnesium concentration with serum testosterone and glucose metabolism marker including fasting insulin (FIN), fasting plasma glucose (FPG), homeostatic model assessment of insulin resistance (HOMA-IR) and quantitative insulin-sensitivity check index (QUICKI) in women with PCOS.

## Materials And Methods

This is a cross-sectional analysis of a large-sample, multi-center, randomized controlled clinical trial in mainland China, with previous publications of protocol and results (16, 17). The institutional review boards at all local sites approved the protocol and all patients signed written informed consent. The trial was registered on ClinicalTrials.gov (NCT01573858) and chictr.org.cn (ChiCTR-TRC-12002081). This trial was designed as a 2 × 2 factorial trial to examine the effects of active acupuncture (or control acupuncture) and clomiphene (or placebo) on live births in anovulatory women with PCOS. All 1000 Participants were diagnosed as PCOS by modified Rotterdam criteria: oligomenorrhea or amenorrhea, together with clinical or biochemical hyperandrogenism (modified Ferriman-Gallwey hirsutism score ≥5 in Chinese) (16, 18), polycystic ovaries, or both (2, 3). Women who took other medications and supplementations recently were excluded.

At baseline visit, physical examination was performed by trained research assistants. Participants will be weighed while dressed in light clothing, without shoes. Waist circumference was measured at the level of the umbilicus and hip circumference was measured at the widest diameter. Height, weight and waist and hip circumferences were recorded to the nearest 0.1 cm, 0.1 kg and 1 cm, respectively. Blood pressure were determined in the right arm in the sitting position. Elevated blood pressures (>=160/100mmHg) would be repeated following a short period of rest. Body mass index (BMI) was calculated by weight (kg)/height (m)^2^.

During baseline visit, all blood samples were collected at day 3 in menstrual cycle then were stored at -80°C and shipped back to the core laboratory at Heilongjiang University of Chinese Medicine for analysis which complied with ISO 15189. Serum magnesium concentration was measured by dimethylamine blue 1 spectrophotometric method in automatic biochemistry analyzer 7600 (Hitachi, Japan). Fasting plasma glucose (FPG) was measured with hexokinase assay (Marker, China), and fasting insulin (FIN) was measured by electro-chemiluminescent immunoassay (Roche Diagnostic, Switzerland). HOMA-IR and QUICKI were calculated by the following formulas: *HOMA-IR=(FIN(mIU/L) × FPG(mmol/L))/22.5* (19); and *QUICKI=1/(log(FIN(mIU/L)) + log(FPG(mg/dL)))* (20). IR status was defined as HOMA-IR >= 2.69 (21). Serum estradiol, total testosterone, luteinizing hormone (LH), follicle-stimulating hormone (FSH) were analyzed by electrochemiluminescent immunoassay (Roche Diagnostic, Switzerland). Sex hormone-binding globulin (SHBG) was measured by immunolite (Simens Diagnostic, Germany). Free androgen index (FAI) was calculated by the following formula: FAI=*total testosterone (nmol/L) × 100/SHBG (nmol/L)* Hyperandrogenemia status was defined as total testosterone >= 1.67 mmol/L, which is the cutoff point in our core laboratory.

### Statistical Analyses

All data were analyzed using Statistical Package for the Social Sciences (SPSS) 24.0 and the results were considered significant if the P value was <0.05. Continuous variables were presented as mean and standard deviation across quartiles of serum magnesium. Analysis of variance was used to compare mineral levels among quartiles of serum magnesium. Pearson correlation analysis was performed to determine possible confounders for the following analysis. Since IR and hyperandrogenemia are also closely related to each other, they are viewed as confounder for each other. Multiple linear regression was used to calculate the adjusted mean with 95% confidence interval (CI) for FIN, FPG, HOMA-IR and QUICKI according to the quartiles of serum magnesium. In model 1, we adjusted for age. In model 2, we additionally adjusted for BMI, systolic blood pressure, diastolic blood pressure, waist circumference and hip circumference. In model 3, we further adjusted for total testosterone, estradiol, FSH, LH, and SHBG. Multiple linear regression was also used to calculate the adjusted mean with 95% CI for total testosterone according to the quartiles of serum magnesium. In model 1, we adjusted for age. In model 2, we additionally adjusted for BMI, waist circumference and hip circumference. In model 3, we further adjusted for FIN and FPG. Trends for associations of serum magnesium concentrations with FIN, FPH, HOMA-IR, QUICKI and testosterone were determined by entering the median value of each quartile and viewing them as a continuous variable by generalized linear model with nominal P values for trend. Logistic regression was performed to investigate the associations between serum magnesium, IR and hyperandrogenemia status, odds ratio (OR) with 95% CI was calculated.

## Results

Of all 1000 women with PCOS, 882 (88.2%) had polycystic ovaries in ultrasound, 584 (58.4%) had hyperandrogenism, of which 442 (44.2%) were biochemical hyperandrogenism. The mean and SD of serum magnesium was 0.87 ± 0.17 mmol/L, and the median and interquartile range was 0.86 (0.76, 0.97) mmol/L. **Table 1** shows the anthropometric and sex hormone characteristics of study participants across quartiles of serum magnesium concentration. Participants with higher quartiles of serum magnesium levels were older. Higher quartiles were also associated with lower FPG, HOMA-IR, total testosterone, LH and FSH, and marginally higher QUICKI. Correlations analyses were demonstrated in **Supplemental Table 1** and variables significantly associated with glucose metabolism and total testosterone were selected as confounders.

**Table 1:** Characteristics of all women with PCOS in quartiles of serum magnesium.

	Q1	Q2	Q3	Q4	P value
Variable					
	228	223	245	243	
Magnesium	0.66 ± 0.08	0.81 ± 0.03	0.91 ± 0.03	1.07 ± 0.11	<0.001
Age (year)	27.5 ± 3.3	27.6 ± 3.3	28.2 ± 3.4	28.2 ± 3.3	0.012
Body mass index (kg/m^2^)	23.8 ± 4.2	24.0 ± 4.3	24.7 ± 4.4	24.1 ± 4.0	0.141
Systolic blood pressure (mmHg)	111.9 ± 10.3	112.6 ± 9.6	112.5 ± 9.0	112.2 ± 8.6	0.845
Diastolic blood pressure (mmHg)	75.3 ± 8.1	75.3 ± 7.6	74.6 ± 8.0	74.2 ± 7.7	0.299
Waist circumference (cm)	84.9 ± 11.9	84.9 ± 11.3	86.1 ± 11.3	84.8 ± 11.3	0.521
Hip circumference (cm)	98.0 ± 8.5	98.3 ± 8.7	98.9 ± 8.8	97.8 ± 8.3	0.489
Glucose (mmol/L)	5.2 ± 1.1	5.2 ± 0.9	5.0 ± 0.9	4.8 ± 0.9	<0.001
Insulin (pmol/L)	105.0 ± 107.5	97.0 ± 79.8	90.4 ± 77.6	97.5 ± 145.3	0.530
HOMA-IR	3.7 ± 4.7	3.4 ± 3.2	3.0 ± 3.0	2.9 ± 2.8	0.029
QUICKI	0.340 ± 0.052	0.336 ± 0.040	0.345 ± 0.047	0.346 ± 0.045	0.078
Estradiol (pmol/L)	289.1 ± 306.7	271.1 ± 299.2	249.5 ± 231.2	263.0 ± 391.5	0.577
Total testosterone (nmol/L)	1.8 ± 0.8	1.7 ± 0.6	1.5 ± 0.6	1.7 ± 0.6	0.001
SHBG (nmol/L)	46.7 ± 30.9	43.7 ± 34.6	40.5 ± 28.6	40.6 ± 28.7	0.096
FAI	6.3 ± 4.9	5.9 ± 4.5	5.6 ± 4.0	5.5 ± 4.1	0.184
Luteinizing hormone (IU/L)	11.7 ± 7.7	11.3 ± 6.4	9.7 ± 5.5	9.8 ± 5.6	0.001
Follicle-stimulating hormone (IU/L)	6.6 ± 2.2	6.3 ± 1.5	5.9 ± 1.5	5.7 ± 1.5	<0.001
LH/FSH	1.9 ± 1.5	1.8 ± 1.0	1. 7 ± 1.0	1.7 ± 1.0	0.338

LH, luteinizing hormone; FSH, follicle stimulating-hormone; HOMA-IR, homeostatic model assessment–insulin resistance; QUICKI, quantitative insulin-sensitivity check index; FAI, free androgen index.

All data were presented as number with mean and standard deviation.

Serum magnesium concentration had significant negative associations with FPG, HOMA-IR, QUICKI and marginally negative association with FIN (**Table 2**). The adjusted means (95% CI) of FIN from the first quartile to the fourth quartile were 102.9 (89.2-116.5) pmol/L, 96.9 (86.3-107.5) pmol/L, 89.7 (80.0-99.4) pmol/L and 89.4 (80.3-98.6) pmol/L after adjusted covariates in model 3 (P for trend = 0.078). Similarly, the values for FPG in four quartiles were 5.26 (5.11-5.40) mmol/L, 5.20 (5.08-5.32) mmol/L, 4.96 (4.84-5.07) mmol/L, 4.76 (4.66-4.87) mmol/L, respectively (P for trend < 0.001). The corresponding values of HOMA-IR were 3.75 (3.13-4.36), 3.38 (2.95-3.81), 3.00 (2.61-3.38), 2.86 (2.50-3.21), respectively (P for trend < 0.001). The corresponding values of QUICKI were 0.341 (0.334-0.347), 0.336 (0.331-0.342), 0.345 (0.339-0.351), 0.347 (0.341-0.352), respectively (P for trend = 0.003).

**Table 2:** Calculated means (95% confidence interval) of insulin, glucose, HOMA-IR, QUICKI across quartiles of serum magnesium after adjusting covariates.

	Q1	Q2	Q3	Q4	Nominal P value for trend^a^
**Fasting insulin**					
Model 1^b^	107.2 (106.7-107.7)	100.3 (100.0-100.5)	95.2 (95.0-95.5)	87.6 (86.9-88.3)	0.401
Model 2^c^	104.3 (99.6-109.0)	98.8 (94.0-103.6)	99.1 (94.6-103.5)	86.9 (82.6-91.2)	0.219
Model 3^d^	102.9 (89.2-116.5)	96.9 (86.3-107.5)	89.7 (80.0-99.4)	89.4 (80.3-98.6)	0.078
**Fasting glucose**					
Model 1^b^	5.26 (5.25-5.28)	5.10 (5.10-5.11)	4.99 (5.00-5.01)	4.82 (4.80-4.84)	<0.001
Model 2^c^	5.25 (5.22-5.28)	5.10 (5.07-5.13)	5.01 (4.99-5.04)	4.82 (4.78-4.85)	<0.001
Model 3^d^	5.26 (5.11-5.40)	5.20 (5.08-5.32)	4.96 (4.84-5.07)	4.76 (4.66-4.87)	<0.001
**HOMA-IR**					
Model 1^b^	3.82 (3.78-3.86)	3.40 (3.37-3.42)	3.12 (3.10-3.15)	2.65 (2.60-2.69)	0.002
Model 2^c^	3.74 (3.57-3.91)	3.34 (3.17-3.50)	3.25 (3.09-3.41)	2.62 (2.46-2.78)	<0.001
Model 3^d^	3.75 (3.13-4.36)	3.38 (2.95-3.81)	3.00 (2.61-3.38)	2.86 (2.50-3.21)	<0.001
**QUICKI**					
Model 1^b^	0.336 (0.335-0.336)	0.340 (0.340-0.341)	0.343 (0.343-0.344)	0.349 (0.348-0.349)	0.044
Model 2^c^	0.338 (0.335-0.341)	0.341 (0.338-0.344)	0.341 (0.339-0.344)	0.349 (0.346-0.352)	0.009
Model 3^d^	0.341 (0.334-0.347)	0.336 (0.331-0.342)	0.345 (0.339-0.351)	0.347 (0.341-0.352)	0.003

HOMA-IR, homeostatic model assessment of insulin resistance; QUICKI, quantitative insulin-sensitivity check index.

a Linear trends across quartiles of serum magnesium were evaluated by entering the median values of each category into the generalized linear model.

b Model 1 adjusted for age.

c Model 2 additionally adjusted for body mass index, waist circumference, hip circumference, systolic blood pressure, diastolic blood pressure.

d Model 3 additionally adjusted for total testosterone, estradiol, follicle-stimulating hormone, luteinizing hormone, and sex hormone-binding globulin.

Serum magnesium concentration also had significant negative associations with total testosterone and marginally negative associations with FAI (**Table 3**). The adjusted means (95% CI) of testosterone from the first quartile to the fourth quartile were 1.73 (1.72-1.74) nmol/L, 1.68 (1.67-1.69) nmol/L, 1.65 (1.64-1.66) nmol/L and 1.59 (1.58-1.60) nmol/L after adjusted covariates in model 3 (P for trend = 0.045). The adjusted means (95% CI) of FAI from the first quartile to the fourth quartile were 6.05 (5.82-6.28), 6.02 (5.79-6.26), 5.63 (5.38-5.87), 5.50 (5.25-5.75) after adjusted covariates in model 3 (P for trend = 0.060).

**Table 3:** Calculated means (95% confidence interval) of testosterone and free androgen index across quartiles of serum magnesium after adjusting covariates.

	Q1	Q2	Q3	Q4	Nominal P value for trend^a^
**Testosterone**					
Model 1^b^	1.73 (1.72-1.73)	1.68 (1.68-1.69)	1.64 (1.64-1.65)	1.59 (1.59-1.60)	0.083
Model 2^c^	1.73 (1.71-1.74)	1.68 (1.67-1.69)	1.65 (1.64-1.66)	1.59 (1.58-1.60)	0.057
Model 3^d^	1.73 (1.72-1.74)	1.68 (1.67-1.69)	1.65 (1.64-1.66)	1.59 (1.58-1.60)	0.045
**Free androgen index**					
Model 1^b^	6.02 (6.00-6.04)	5.85 (5.84-5.86)	5.76 (5.75-5.77)	5.61 (5.60-5.63)	0.093
Model 2^c^	5.98 (5.76-6.20)	6.04 (5.80-6.27)	5.67 (5.43-5.92)	5.49 (5.25-5.74)	0.078
Model 3^d^	6.05 (5.82-6.28)	6.02 (5.79-6.26)	5.63 (5.38-5.87)	5.50 (5.25-5.75)	0.060

a Linear trends across quartiles of serum magnesium were evaluated by entering the median values of each category into the generalized linear model.

b Model 1 adjusted for age.

c Model 2 additionally adjusted for body mass index, waist circumference, hip circumference.

d Model 3 additionally adjusted for estradiol, insulin and glucose.

Logistic regression showed that serum magnesium in quartile 1 and 2 were marginally associated with IR (**Table 4**) after adjusting age. After additionally adjusting BMI, waist circumference, hip circumference, systolic blood pressure, diastolic blood pressure, serum magnesium in quartile 1 and 2 were significantly associated with IR status (Quartile 1: OR: 1.75, 95%CI: 1.13-2.72, P=0.012; Quartile 2: OR: 1.67, 95%CI: 1.07-2.60, P=0.023). After further adjusting total testosterone, estradiol, FSH, LH, and SHBG, serum magnesium in quartile 1 and 2 were still significantly associated with IR status (Quartile 1: OR: 2.15, 95%CI: 1.35-3.40, P=0.001; Quartile 2: OR: 1.90, 95%CI: 1.20-3.02, P=0.006).

**Table 4:** Association between insulin resistance status and serum magnesium according to quartiles by logistic regression.

	Insulin resistance	OR in model 1^a^	P value	OR in model 2^b^	P value	OR in model 3^c^	P value
**Serum magnesium**							
Quartile 1	103 (46.2%)	1.44 (0.99-2.10)	0.054	1.75 (1.13-2.72)	0.012	2.15 (1.35-3.40)	0.001
Quartile 2	102 (46.4%)	1.45 (1.00-2.11)	0.050	1.67 (1.07-2.60)	0.023	1.90 (1.20-3.02)	0.006
Quartile 3	98 (40.3%)	1.14 (0.79-1.65)	0.485	1.02 (0.67-1.56)	0.930	1.09 (0.71-1.69)	0.684
Quartile 4	89 (37.4%)	1 (reference)		1 (reference)		1 (reference)	

a Model 1 adjusted for age.

b Model 2 additionally adjusted for body mass index, waist circumference, hip circumference, systolic blood pressure, diastolic blood pressure.

c Model 3 additionally adjusted for total testosterone, estradiol, follicle-stimulating hormone, luteinizing hormone, and sex hormone-binding globulin.

For association between serum magnesium and hyperandrogenemia (**Table 5**), serum magnesium in quartiles were not associated with hyperandrogenemia status in model 1. After additionally adjusting BMI, waist circumference, hip circumference, serum magnesium in quartile 1 was marginally associated with IR status (Quartile 1: OR: 1.38, 95%CI: 0.96-2.00, P=0.085). After further adjusting FIN and FPG, serum magnesium in quartile 1 were still marginally associated with hyperandrogenemia status (Quartile 1: OR: 1.45, 95%CI: 0.99-2.11, P=0.055). **Supplemental Tables 2**, **3** detect whether IR and hyperandrogenemia had a modifier effect for each other. For women with and without hyperandrogenemia, associations between serum magnesium and IR were consistent with that of all women. Similarly, for women with and without IR, associations between serum magnesium and hyperandrogenemia were consistent with that in all women.

**Table 5:** Association between hyperandrogenemia and serum magnesium according to quartiles by logistic regression.

	Hyperandrogenemia	OR in model 1^a^	P value	OR in model 2^b^	P value	OR in model 3^c^	P value
**Serum magnesium**							
Quartile 1	119 (52.7%)	1.35 (0.94-1.95)	0.106	1.38 (0.96-2.00)	0.085	1.45 (0.99-2.11)	0.055
Quartile 2	108 (48.9%)	1.17 (0.81-1.69)	0.411	1.19 (0.82-1.72)	0.361	1.19 (0.81-1.73)	0.370
Quartile 3	93 (38.0%)	0.75 (0.52-1.08)	0.123	0.74 (0.51-1.06)	0.100	0.72 (0.50-1.04)	0.080
Quartile 4	107 (44.6%)	1 (reference)		1 (reference)		1 (reference)	

a Model 1 adjusted for age.

b Model 2 additionally adjusted for body mass index, waist circumference, hip circumference.

c Model 3 additionally adjusted for estradiol, insulin and glucose.

## Discussion

This study investigated the association between serum magnesium concentration, IR, and testosterone level in Chinese women with PCOS. Our results demonstrated that serum magnesium concentration had independent negative associations with FIN, FPG, HOMA-IR, testosterone and positive association with insulin sensitivity index QUICKI.

Hyperandrogenemia and IR both played an irreplaceable role in the pathogenesis of PCOS, although the underlying mechanism are still not clearly understood. Gene factors such as polymorphisms in androgen receptor gene and follistatin gene might contribute to hyperandrogenemia in women of PCOS, while genetic change in melatonin receptor 1B gene may impair insulin secretion and increase FPG (4). IR and hyperandrogenemia are also closely related to each other. Obesity might exacerbate the progress of IR and hyperandrogenemia in women with PCOS. Women with PCOS in this study are in much lower BMI compared to Caucasian women with PCOS in two randomized controlled trials (22, 23). Our results suggested that lower serum magnesium might independently contribute to the formation of IR and hyperandrogenemia in women with PCOS.

In the present study, lower serum magnesium concentration was associated with higher FPG, HOMA-IR, and lower QUICKI, which was consistent with previous studies in general populations. Kieboom et al. conducted a population-based cohort study of 8555 male and female participants with normal glucose levels and found that a 0.1 mmol/l decrease in serum magnesium level was associated with an increase in diabetes risk (hazard ratio 1.18 [95% CI 1.04, 1.33]) (24). Another study in prediabetic or early untreated diabetic Chinese postmenopausal women indicated lower serum magnesium is significantly associated with IR (25). Hruby et al. found that higher magnesium intake was associated with lower risk of prediabetes and/or IR, and progression from these states to type 2 diabetes in 2,582 community-dwelling participants (26).

Serum magnesium is a cofactor for many enzymes involved in multiple biological functions. However, the mechanism between serum magnesium, IR and diabetes mellitus are still not completely understood. Magnesium might affect insulin secretion, peripheral insulin sensitivity, insulin signaling, insulin-signaling kinases, low-grade systemic inflammation, carbohydrate and energy Metabolism, which lead to IR and diabetes mellitus (27, 28). Our results demonstrated that serum magnesium had no strong association with FIN, suggesting serum might had small effect on insulin secretion. However, serum magnesium had very obvious associations with FPG, HOMA-IR and QUICKI, indicating lower serum magnesium might aggravate IR by regulating insulin sensitivity.

Our results also showed that lower serum magnesium was associated with higher serum testosterone in women with PCOS. One study found that magnesium supplementation increased free and total testosterone levels in male athletes (29). However, few studies have explored the association between serum magnesium concentration and testosterone in women. In normal cycling women, ionized Mg was negatively associated with testosterone level (30), while no relationship was found between magnesium and testosterone in women after menopause (31). As well, one study showed that serum magnesium could uncompetitively inhibit the binding between testosterone and SHBG, therefore influence the bioavailability of testosterone (32).

Magnesium is an antioxidant and acts as a cofactor for several enzymes, which maintains cell membrane stability and mitigates the effects of oxidative stress (33). Oxidative stress is related to IR and hyperandrogenism in women with PCOS. Evidence suggested that antioxidants such as coenzyme Q10 or Vitamin E for 8 weeks led to decreased HOMA-IR and serum total testosterone levels compared with those of the placebo group (34). Therefore, antioxidant effect of serum magnesium might be the mechanism to decrease IR and testosterone level in women with PCOS.

Based on our results, it is possible that magnesium supplementation can alleviate the severity of PCOS or prevent the onset of PCOS. Elderawi et al. conducted a randomized controlled trial and revealed that oral magnesium supplementation reduces IR and improves the glycemic control indicators among type 2 diabetes patients (35). Some randomized controlled trials with small sample size demonstrated multiple nutrition supplementation including magnesium had benefits in improving insulin metabolism, cardiometabolic profiles and oxidative stress for women with PCOS (36–39). More prospective larger trials are needed to confirm the benefits of screening serum magnesium status and magnesium supplementation for women with PCOS in clinical practice.

The major strengths of the present study are large sample size and potential confounding variables were adjusted. Furthermore, participants were recruited in more than twenty local sites and had representativeness of Chinese women with PCOS. The limitation of the present study was that it was not designed to examine the research question, therefore the sample size was not calculated for the association between serum magnesium, IR and testosterone. Second, we did not use the golden standard glucose clamp to evaluate IR. Furthermore, our participants were limited mostly in Chinese Han women with PCOS, and more studies in different cohort of women with PCOS are needed.

## Conclusions

In conclusion, lower serum magnesium was associated with glucose metabolism including FIN, FPG, HOMA-IR, QUICKI and higher testosterone level in women with PCOS. More larger trials are required to confirm the benefits of screening magnesium status and magnesium supplementation on IR and testosterone in women with PCOS.

## Data Availability Statement

The original contributions presented in the study are included in the article/**Supplementary Material**. Further inquiries can be directed to the corresponding authors.

## Ethics Statement

The studies involving human participants were reviewed and approved by First Affiliated Hospital, Heilongjiang University of Chinese Medicine. The patients/participants provided their written informed consent to participate in this study.

## Author Contributions

X-KW designed the study and critically revised the manuscript. W-YC and XL performed data analysis. W-YC, XL, X-MY, H-LM, JC, HC, J-SG, W-JS, and YW collected data. W-YC and XL drafted the manuscript. All authors contributed to the article and approved the submitted version.

## Funding

National Public Welfare Projects for Chinese Medicine (201107005, 200807002), the National Key Discipline of Chinese Medicine in Gynecology during the year of 2009–2016 (JC200804), the Intervention for Polycystic Ovary Syndrome Based on Traditional Chinese Medicine Theory—‘Tian Gui Disorder’ (2011TD006), and the National Clinical Trial Base in Chinese Medicine Special Projects (JDZX2012036, 2015B009) during the year of 2009–2016 for the First Affiliated Hospital, Heilongjiang University of Chinese Medicine, as well as the Heilongjiang Province ‘Longjiang Scholar’ Program to X-KW. Heilongjiang Natural Science Foundation (H2018051).

## Conflict of Interest

The authors declare that the research was conducted in the absence of any commercial or financial relationships that could be construed as a potential conflict of interest.
