# Supplementary material for: Associations of Serum Magnesium With Insulin Resistance and Testosterone in Women With Polycystic Ovary Syndrome
Source: Front Endocrinol (Lausanne). 2021 Jun 23;12:683040. doi: 10.3389/fendo.2021.683040 (PMC8261149; doi:10.3389/fendo.2021.683040)
Supplement: Supplementary file 1 [file Table_1.docx]

| **Supplemental Table 1 Correlation analyses between possible confounders, serum magnesium, glucose metabolism and total testosterone** | | | | | | |
| --- | --- | --- | --- | --- | --- | --- |
|  | Serum magnesium | Glucose | Insulin | HOMA-IR | QUICKI | Total testosterone |
| Age | .100^**^ | 0.021 | -0.015 | 0.029 | -0.014 | -.071^*^ |
| Body mass index | 0.000 | .192^**^ | .335^**^ | .350^**^ | -.439^**^ | 0.163^*^ |
| Systolic blood pressure | 0.002 | .136^**^ | .136^**^ | .135^**^ | -.198^**^ | 0.076 |
| Diastolic blood pressure | -0.047 | .125^**^ | .083^**^ | .092^**^ | -.129^**^ | 0.054 |
| Waist circumference | -0.019 | .196^**^ | .299^**^ | .342^**^ | -.421^**^ | 0.149^*^ |
| Hip circumference | -0.029 | .177^**^ | .264^**^ | .272^**^ | -.402^**^ | 0.115^*^ |
| Estradiol | -0.033 | 0.085^*^ | -0.044 | -0.031 | 0.043 | .226^**^ |
| SHBG | -0.055 | -0.042 | -.188^**^ | -.197^**^ | .222^**^ | -0.022 |
| Luteinizing hormone | -.120^**^ | 0.009 | -.147^**^ | -.134^**^ | .141^**^ | .310^**^ |
| Follicle-stimulating hormone | -.220^**^ | .070^*^ | -.071^*^ | -0.054 | 0.044 | -0.036 |
| LH/FSH | -0.044 | -0.016 | -.113^**^ | -.103^**^ | .108^**^ | .338^**^ |
| SHBG (sex hormone-binding globulin), HOMA-IR (homeostatic model assessment–insulin resistance), QUICKI (quantitative insulin-sensitivity check index), LH (luteinizing hormone), FSH (follicle stimulating-hormone),  All data were presented as Pearson correlation coefficient.  ^*^ P<0.05, ^**^ P<0.01 | | | | | | |

| **Supplemental Table 2 Association between insulin resistance status and serum magnesium according to quartiles stratified by hyperandrogenemia** | | | | | | | |
| --- | --- | --- | --- | --- | --- | --- | --- |
|  | Insulin resistance | OR in model 1 ^a^ | P value | OR in model 2 ^b^ | P value | OR in model 3 ^c^ | P value |
| ***With***  ***hyperandrogenemia*** |  |  |  |  |  |  |  |
| **Serum magnesium** |  |  |  |  |  |  |  |
| Quartile 1 | 56 (47.5%) | 1.26 (0.74-2.14) | 0.399 | 1.86 (0.97-3.56) | 0.061 | 2.23 (1.12-4.42) | 0.022 |
| Quartile 2 | 50 (46.7%) | 1.22 (0.71-2.11) | 0.469 | 1.69 (0.88-3.24) | 0.118 | 2.07 (1.03-4.17) | 0.041 |
| Quartile 3 | 34 (37.0%) | 0.84 (0.48-1.50) | 0.563 | 0.90 (0.46-1.79) | 0.768 | 0.98 (0.49-1.97) | 0.950 |
| Quartile 4 | 44 (41.5%) | 1 (reference) |  | 1 (reference) |  | 1 (reference) |  |
| ***Without hyperandrogenemia*** |  |  |  |  |  |  |  |
| **Serum magnesium** |  |  |  |  |  |  |  |
| Quartile 1 | 47 (44.8%) | 1.60 (0.94-2.71) | 0.083 | 1.72 (0.94-3.16) | 0.081 | 2.10 (1.10-4.02) | 0.024 |
| Quartile 2 | 52 (46.0%) | 1.68 (1.00-2.81) | 0.051 | 1.78 (0.97-3.25) | 0.064 | 1.95 (1.03-3.67) | 0.039 |
| Quartile 3 | 64 (42.4%) | 1.43 (0.88-2.33) | 0.146 | 1.12 (0.64-1.95) | 0.696 | 1.21 (0.68-2.13) | 0.520 |
| Quartile 4 | 45 (34.1%) | 1 (reference) |  | 1 (reference) |  | 1 (reference) |  |
| ^a^ Model 1 adjusted for age  ^b^ Model 2 additionally adjusted for body mass index, waist circumference, hip circumference, systolic blood pressure, diastolic blood pressure  ^c^ Model 3 additionally adjusted for total testosterone, estradiol, follicle-stimulating hormone, luteinizing hormone, and sex hormone-binding globulin | | | | | | | |

| **Supplemental Table 3 Association between hyperandrogenemia and serum magnesium according to quartiles stratified by insulin resistance** | | | | | | | |
| --- | --- | --- | --- | --- | --- | --- | --- |
|  | Hyperandrogenemia | OR in model 1 ^a^ | P value | OR in model 2 ^b^ | P value | OR in model 3 ^c^ | P value |
| ***With***  ***insulin resistance*** |  |  |  |  |  |  |  |
| **Serum magnesium** |  |  |  |  |  |  |  |
| Quartile 1 | 56 (54.4%) | 1.15 (0.65-2.04) | 0.635 | 1.17 (0.66-2.09) | 0.587 | 1.17 (0.99-1.38) | 0.058 |
| Quartile 2 | 50 (49.0%) | 0.93 (0.52-1.65) | 0.804 | 0.94 (0.53-1.67) | 0.831 | 0.89 (0.49-1.60) | 0.687 |
| Quartile 3 | 34 (34.7%) | 0.70 (0.40-1.23) | 0.110 | 0.75 (0.32-1.31) | 0.130 | 0.70 (0.27-1.81) | 0.103 |
| Quartile 4 | 44 (49.4%) | 1 (reference) |  | 1 (reference) |  | 1 (reference) |  |
| ***Without***  ***insulin resistance*** |  |  |  |  |  |  |  |
| **Serum magnesium** |  |  |  |  |  |  |  |
| Quartile 1 | 62 (51.7%) | 1.49 (0.92-2.42) | 0.106 | 1.56 (0.96-2.54) | 0.076 | 1.55 (0.94-2.56) | 0.060 |
| Quartile 2 | 57 (48.3%) | 1.31 (0.80-2.13) | 0.282 | 1.40 (0.86-2.30) | 0.178 | 1.41 (0.86-2.33) | 0.176 |
| Quartile 3 | 58 (40.0%) | 0.92 (0.58-1.47) | 0.726 | 0.92 (0.58-1.48) | 0.738 | 0.92 (0.57-1.47) | 0.721 |
| Quartile 4 | 62 (41.6%) | 1 (reference) |  | 1 (reference) |  | 1 (reference) |  |
| ^a^ Model 1 adjusted for age  ^b^ Model 2 additionally adjusted for body mass index, waist circumference, hip circumference  ^c^ Model 3 additionally adjusted for estradiol, insulin and glucose | | | | | | | |
